# Electroplated core–shell nanowire network electrodes for highly efficient organic light-emitting diodes

**DOI:** 10.1186/s40580-021-00295-2

**Published:** 2022-01-05

**Authors:** Hyungseok Kang, Joo Sung Kim, Seok-Ryul Choi, Young-Hoon Kim, Do Hwan Kim, Jung-Gu Kim, Tae-Woo Lee, Jeong Ho Cho

**Affiliations:** 1 SKKU Advanced Institute of Nanotechnology (SAINT), Sungkyunkwan University, Suwon, 440-746 Republic of Korea; 2 School of Advanced Materials Science and Engineering, Sungkyunkwan University, Suwon, 440-746 Republic of Korea; 3 Department of Materials Science and Engineering, Seoul National University, Seoul, 08826 Republic of Korea; 4 Department of Chemical Engineering, Hanyang University, Seoul, 04763 Republic of Korea; 5 School of Chemical and Biological Engineering, Research Institute of Advanced Materials, Institute of Engineering Research, Nano Systems Institute (NSI), BK21 PLUS SNU Materials Division for Educating Creative Global Leaders, Seoul National University, Seoul, 08826 Republic of Korea; 6 Department of Chemical and Biomolecular Engineering, Yonsei University, Seoul, 03722 Republic of Korea

**Keywords:** Metal nanowire, Organic light-emitting diode, Electroplating, Work function, Transparent electrode

## Abstract

In this study, we performed metal (Ag, Ni, Cu, or Pd) electroplating of core–shell metallic Ag nanowire (AgNW) networks intended for use as the anode electrode in organic light-emitting diodes (OLEDs) to modify the work function (WF) and conductivity of the AgNW networks. This low-cost and facile electroplating method enabled the precise deposition of metal onto the AgNW surface and at the nanowire (NW) junctions. AgNWs coated onto a transparent glass substrate were immersed in four different metal electroplating baths: those containing AgNO_3_ for Ag electroplating, NiSO_4_ for Ni electroplating, Cu_2_P_2_O_7_ for Cu electroplating, and PdCl_2_ for Pd electroplating. The solvated metal ions (Ag^+^, Ni^2+^, Cu^2+^, and Pd^2+^) in the respective electroplating baths were reduced to the corresponding metals on the AgNW surface in the galvanostatic mode under a constant electric current achieved by linear sweep voltammetry via an external circuit between the AgNW networks (cathode) and a Pt mesh (anode). The amount of electroplated metal was systematically controlled by varying the electroplating time. Scanning electron microscopy images showed that the four different metals (shells) were successfully electroplated on the AgNWs (core), and the nanosize-controlled electroplating process produced metal NWs with varying diameters, conductivities, optical transmittances, and WFs. The metal-electroplated AgNWs were successfully employed as the anode electrodes of the OLEDs. This facile and low-cost method of metal electroplating of AgNWs to increase their WFs and conductivities is a promising development for the fabrication of next-generation OLEDs.

**Supplementary Information:**

The online version contains supplementary material available at 10.1186/s40580-021-00295-2.

## Introduction

The replacement of indium tin oxide (ITO) with metal nanowires (e.g. silver nanowires (AgNWs), copper nanowires, gold nanowires) in the fabrication of transparent conductive electrodes (TCEs) has been investigated in the past decades [1–6]. The AgNW TCE has been utilized not only in optoelectronic devices, such as solar cells, light-emitting diodes (LEDs), and flexible displays, but also in wearable devices, such as e-skin sensors and energy-harvesting ferroelectric/triboelectric nanogenerators [7–10]. Although carbon nanomaterials (such as graphene and carbon nanotubes) and conducting polymers have been in the spotlight as possible replacements for ITO, their low electrical conductivities restrict their practical application in a variety of devices [11, 12]. AgNWs are the most promising TCE material because of their excellent conductivity in addition to their high optical transparency, high mechanical flexibility, and simple fabrication process for AgNWs network [13–15]. Notably, AgNWs can be dispersed in a variety of organic solvents; as a result, various processes can be used to fabricate TCEs, such as the Meyer-rod coating, spray-coating, and spin-coating methods [16–19]. Despite the great potential of AgNWs to be used as a TCE material, their use as the anode electrode of organic LEDs (OLEDs) has been limited by the low work function (WF ~ 4.3 eV) and high electrical resistance of the as-coated AgNW electrode (several tens of ohms at an optical transmittance of 80%) [20–22]. The relatively low WF of the AgNW anode causes difficulty in hole injection from the anode to the organic layer because of a high barrier potential at the junction (for instance, the ionization potential of Super Yellow is 5.2 eV) [23]. As a result, multiple hole injection layers (HILs) e.g., poly (3,4-ethylenedioxythiophene): poly(4-styrene sulfonate) (PEDOT:PSS) and gradient HIL composed of PEDOT:PSS and a perfluorinated ionomer for use as an anode and electron injection layer(e.g. polyethylenimine (PEI)) for use as a cathode have been necessary to operate OLEDs based on the AgNW anode [24–26]. Furthermore, several post-welding methods for minimizing the contact resistance at the NW junctions have been explored with the aim of reducing the high sheet resistance of the as-coated AgNW electrode. Examples of such methods include welding using heat energy (e.g., thermal welding, plasmonic welding, and Joule heating), chemical reduction, and electrochemical metal deposition [27–38].

To address both these issues (low WF and high sheet resistance of the AgNW electrode) simultaneously, we employed a nanoscale metal (M = Ag, Ni, Cu, or Pd) electroplating technique for fabricating metal-electroplated core–shell AgNW (M-AgNW) network electrodes. Specifically, the Ni electroplating for AgNWs network which is low-cost and facile electroplating technique not only increased the WF of the AgNW electrode, but also reduced its sheet resistance. AgNWs deposited onto a transparent glass substrate were directly immersed in various electroplating baths: those containing AgNO_3_ for Ag electroplating, NiSO_4_ for Ni electroplating, Cu_2_P_2_O_7_ for Cu electroplating, and PdCl_2_ for Pd electroplating. The metal ions solvated in their respective electroplating baths were reduced to the corresponding metals on the AgNW surface by the application of an electric current through an external circuit between the AgNW network (cathode) and a Pt mesh (anode). The electric current was generated by linear sweep voltammetry (LSV) of each metal element, and the amount of electroplated metal was systematically controlled by varying the electroplating time. The metal (shell) was successfully electroplated on the AgNWs (core), and the NW diameters, electrical conductivities, optical transmittances, and WFs of the M-AgNWs were precisely controlled. Unlike our previous study [39], various metals with higher WFs compared with Ag were electroplated onto AgNWs and their growth mechanism was systematically investigated. The M-AgNW electrode was successfully applied in a Super Yellow OLED. The device with a high-WF and low-resistance Ni-AgNW anode exhibited even higher efficiency (11.60 cd/A, 7.90 lm/W and 4.63%) than an OLED with the conventional ITO anode (9.51 cd/A, 4.05 lm/W and 3.80%). This simple and low-cost metal electroplating method for adjusting the WFs and conductivities of the AgNW electrodes has great potential to be used in the fabrication of next-generation optoelectronic devices.

## Experimental section

### Metal electroplating process

The Ag electroplating solution contained 0.4 g/L of AgNO_3_, 4 g/L of K_2_S_2_O_5_, 22.5 g/L of Na_2_S_2_O_3_, and CH_3_COONH_4_, and its pH ranged between 5.5 and 6. The Ni electroplating solution contained 150 g/L of NiSO_4_, 15 g/L of NH_4_Cl, and 15 g/L of H_3_BO_3_, and its pH ranged between 5.5 and 6. The Cu electroplating solution contained 80 g/L of Cu_2_P_2_O_7_, 290 g/L of K_4_P_2_O_7_, and 3 g/L of NH_3_, and its pH ranged between 5.5 and 6. The Pd electroplating solution contained 1 g/L of PdCl_2_, 11.6 g/L of NaCl, and 2 g/L of NaNO_3_. A two-electrode cell (500 mL) was used for electrodeposition, wherein the AgNW cathode was prepared with a dispersion of 0.5 wt% AgNWs in isopropyl alcohol (Nanopyxis Co.; AgNW diameter: ~ 30 nm; and length: ~ 30 μm) deposited onto a glass substrate by the Meyer-rod coating method (rod #7) and the anode was a Pt mesh. The prepared AgNW network film (working electrode) was immersed in the metal (Ag, Ni, Cu or Pd) electroplating bath at a constant current density (2 mA/cm^2^). LSV was performed at a controlled electrode rotation speed (500 rpm). The electrode potential was linearly swept from − 0.8 V to − 2.5 V *versus* the Pt electrode at a potential sweep rate of 20 mV/s. The pristine AgNW film and electroplated core–shell NW (Ag-AgNW, Ni-AgNW, Cu-AgNW, and Pd-NW) films were visualized by SEM (JSM-7600F, JEOL, Ltd.) and the work function data of each core–shell nanowires was scanned by 15 eV using UPS microprobe (ESCALAB 250, Thermo Fisher). The sheet resistance was measured by the four-point probe technique (Keithley 2182A and 6221), and the optical transmittance was measured using a UV–vis spectrophotometer (V-650, Jasco).

### OLED fabrication

First, a 50-nm-thick PEDOT:PSS (Clevios AI4083) layer was coated onto the M-AgNW-deposited glass substrate by spin coating at 2000 rpm, which was followed by annealing at 150 °C for 20 min; then, the resultant structure was transferred to an N_2_-filled glove box. Super Yellow (PDY-132, Merck) dissolved in toluene (0.9 wt%) was spin-cast onto the HIL at 4500 rpm to obtain an 80-nm-thick emitting layer. The resultant structure was annealed at 80 °C for 20 min and then loaded into an ultra-high vacuum chamber (~ 10^–7^ Torr) to thermally deposit LiF (1 nm) and Al (100 nm) as the cathode. The fabricated OLEDs were encapsulated with a glass lid by means of epoxy resin in an N_2_ atmosphere. An integrated measurement system comprising a Keithley 236 source measurement unit and a Konica-Minolta CS-2000 spectroradiometer was used to measure the current density (*J*)–voltage (*V*)–luminance (*L*) characteristics of the devices.

## Results and discussion

**Fig. 1:**
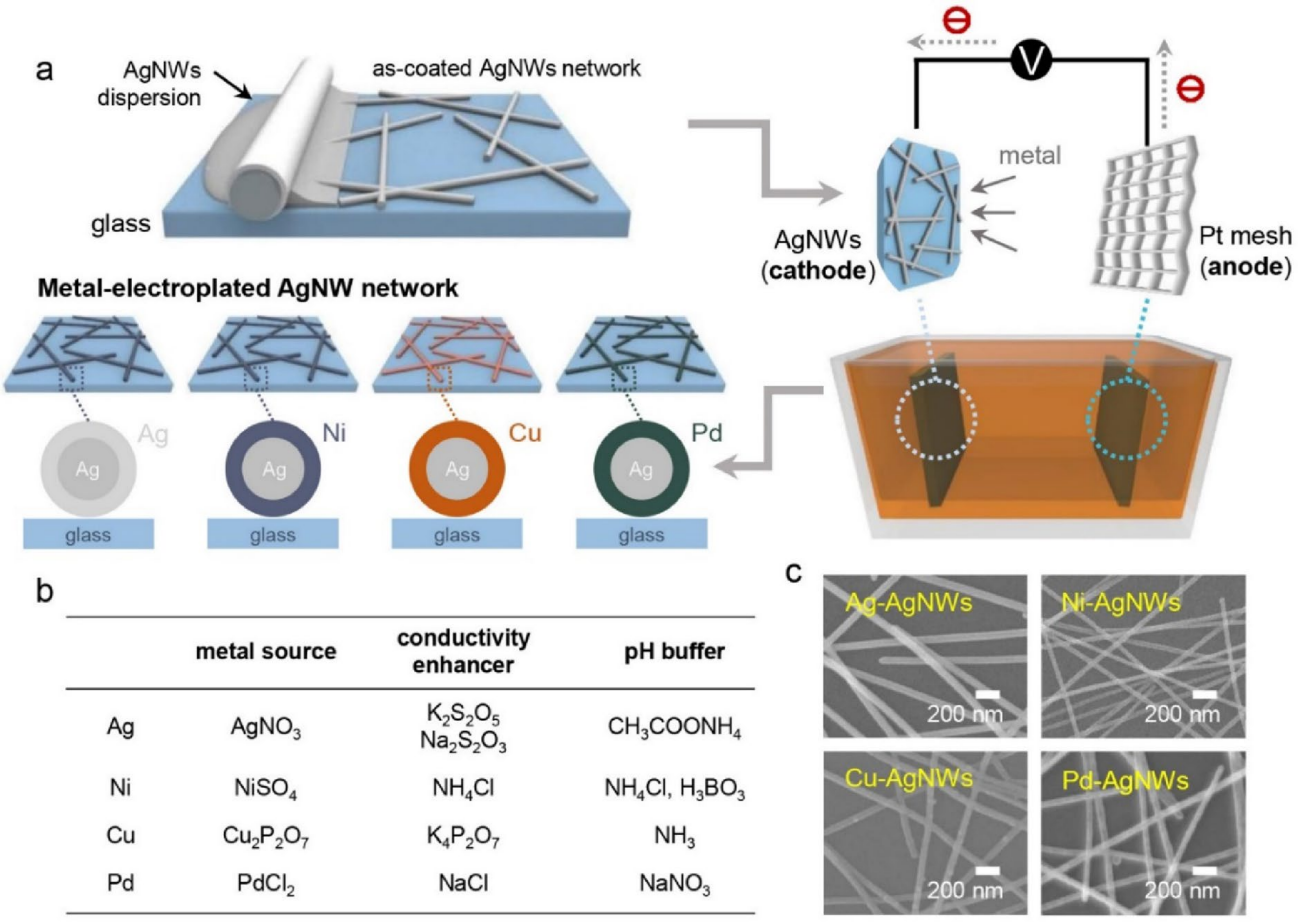
**a** Schematic illustration of the process of metal electroplating of the AgNW network electrode. **b** List of constituents of various metal electroplating baths. **c** SEM images of metal-electroplated AgNW electrodes

A schematic of the fabrication procedure of the metal-electroplated AgNW network is shown in Fig. 1a. First, AgNWs were deposited onto a glass substrate by the Meyer-rod coating method using rod #7 (Additional file 1: Fig. S1). The as-coated AgNWs, which were used as the cathode electrode, were connected to a Pt mesh anode electrode through an external circuit for the electroplating process. To control electroplating precisely, Pt mesh with inert and high exchange current density property was utilized as anode electrode, preventing slow oxidation reaction from low exchange current density property which can result in difficulty on controlling the growth rate of electroplating [40–42]. Four different metal (Ag, Ni, Cu, and Pd) electroplating solutions were prepared; their compositions are shown in Fig. 1b [43–45]. The electroplating solutions essentially have three components: a metal source, conductivity enhancer, and pH buffer. The primary metal source provided metal ions for the deposition of the metal element onto the AgNW surface; the conductivity enhancer enabled rapid movement of the metal ions in the electroplating solution; and the pH buffer had the important function of suppressing the decrease in pH of the solution. In our approach, the AgNWs were soluble in the electroplating solution as the Ag element preferentially exists as Ag^+^ at pH 5 or lower, according to thermodynamics [46–49]. The Ag electroplating solution was composed of AgNO_3_ (metal source), K_2_S_2_O_5_ and Na_2_S_2_O_3_ (conductivity enhancer), and CH_3_COONH_4_ (pH buffer). The Ni electroplating solution was composed of NiSO_4_^¯^ (metal source), NH_4_Cl (conductivity enhancer and pH buffer), and H_3_BO_3_ (pH buffer). The Cu electroplating solution was composed of Cu_2_P_2_O_7_ (metal source), K_4_P_2_O_7_ (conductivity enhancer), and NH_3_ (pH buffer). The Pd electroplating solution was composed of PdCl_2_ (metal source), NaCl (conductivity enhancer), and NaNO_3_ (pH buffer). The as-coated AgNW electrode cathode and Pt mesh electrode anode were located at fixed positions to maintain a distance between them with the aim of providing a uniform current density during the electroplating process. Note that.

Figure 1c shows the scanning electron microscopy (SEM) images of the electroplated NWs (electroplating conditions: current of 30 mA and electroplating time of 8 s). The electroplating conditions, including the voltage, current density, and electroplating time, were smartly controlled to optimize the optoelectronic properties (NW diameter, sheet resistance, and optical transmittance) of the AgNW network film.

**Fig. 2:**
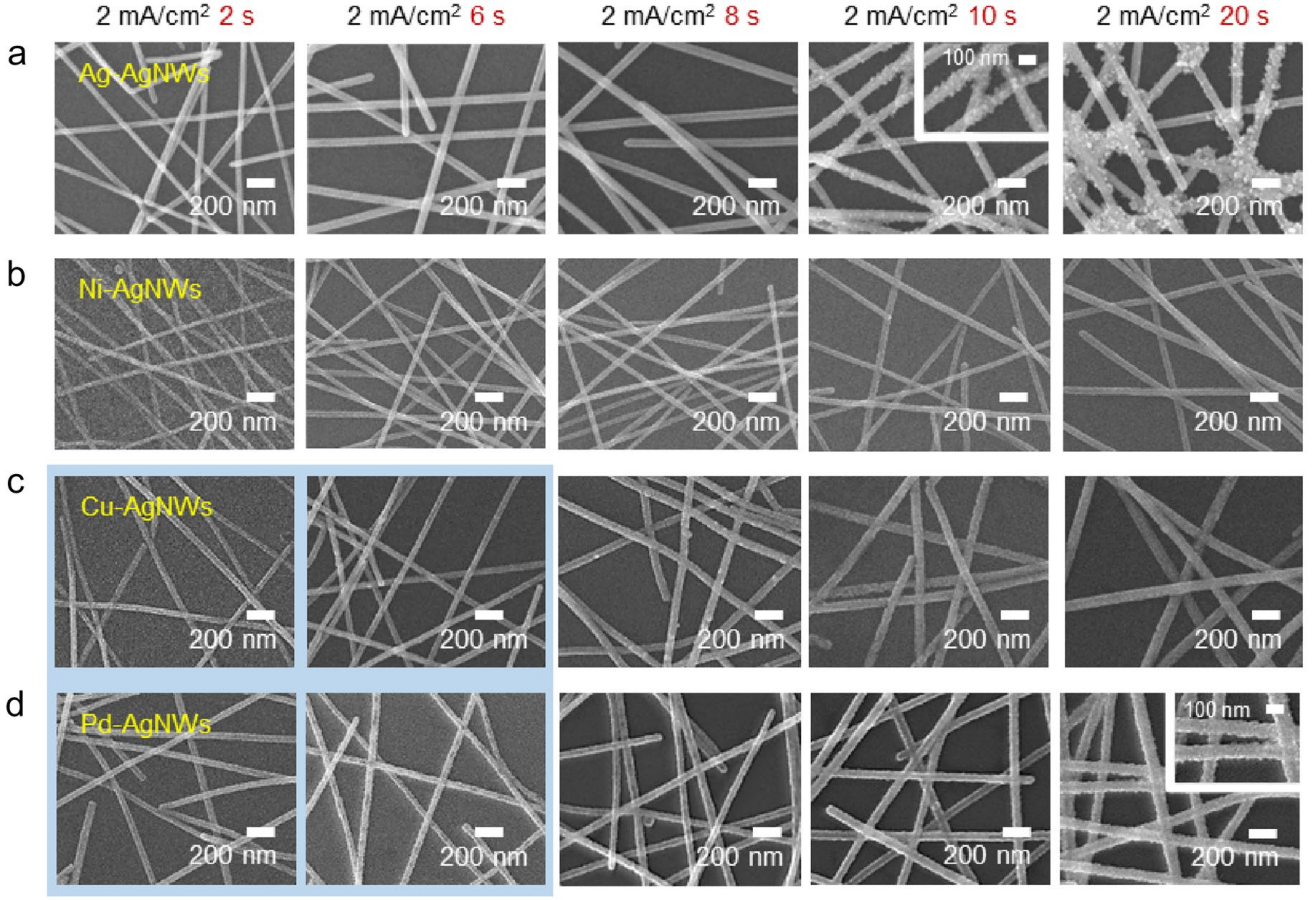
SEM images of **a** Ag-AgNW, **b** Ni-AgNW, **c** Cu-AgNW, and **d** Pd-AgNW films at various electroplating times (2, 6, 8, 10, and 20 s) at a constant current density of 2 mA/cm^2^

The surface morphology of the M-AgNW electrodes was observed by the scanning electron microscopy (SEM), as shown in Fig. 2. All four electroplating processes (i.e., with the four different metals) were performed under the same electroplating conditions with electroplating times ranging from 2 to 20 s and at a constant current density of 2 mA/cm^2^. Figure 2a shows the SEM images of the Ag-electroplated AgNWs (Ag-AgNWs). The surfaces of the AgNWs were smooth and clean upon the application of current for up to 8 s. However, after 8 s (i.e., current application for 10 s and 20 s), their surfaces became rough and contained numerous aggregated Ag particles. The SEM images of the Ni-electroplated AgNWs (Ni-AgNWs) shown in Fig. 2b indicate that the NW surface was relatively cleaner and the Ni-AgNWs had a smaller diameter (58 nm at 20 s) than that of the Ag-AgNWs. Figures 2c and d show the SEM images of the Cu-AgNWs and Pd-AgNWs, respectively. The surface morphologies of both these electroplated AgNWs were relatively smoother compared to that of the Ag-AgNWs. However, the Cu-AgNWs also had a rough NW surface and small particles were formed on the surface of the Pd-AgNWs. The uneven plating of both Cu and Pd on the AgNW surface could have been a result of the concentration gradient of the solution. Particle formation during the Cu, Pd, and Ag electroplating was attributed to the fact that the electroplating rates of all these metals were faster than that of Ni, which created a non-uniform concentration gradient of metal ions near the AgNW surface.

**Fig. 3:**
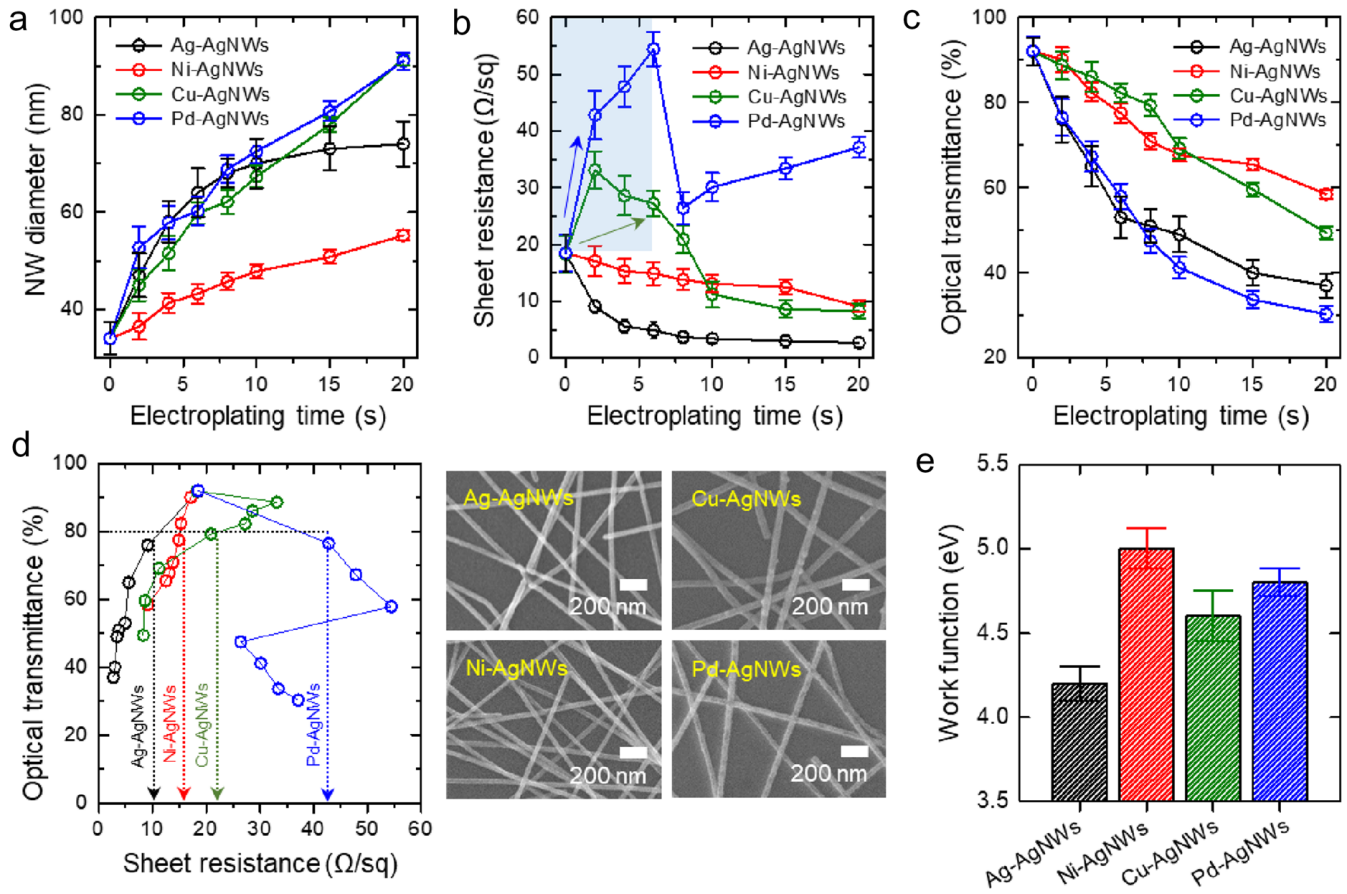
**a** NW diameter **b** sheet resistance **c** optical transmittance at 550 nm of M-AgNW films as functions of electroplating time at a constant current density of 2 mA/cm^2^ **d** optical transmittance at 550 nm of M-AgNW films as a function of sheet resistance. The right panel shows the SEM images of the M-AgNW films used for OLED fabrication. **e** WFs of M-AgNW films

The diameters of the electroplated NWs are plotted as a function of the electroplating time as shown in Fig. 3a. Generally, the NW diameter is proportional to the square root of the electroplating time. That is, the amount of plated metal is linearly proportional to the electroplating time and the increment rate of the NW diameter gradually decreases with increasing electroplating time because of the equality between the cross-sectional area of the deposited NWs and the square of the diameter [34, 50]. Under the same electroplating conditions (electroplating time of 20 s and applied current density of 2 mA/cm^2^), the Ni-AgNWs had a diameter of 58 nm, Ag-AgNWs had a diameter of 72 nm, and Cu-AgNWs and Pd-AgNWs had a diameter of 90 nm each. That is, despite the identical current applied to the AgNWs, the diameters of the plated NWs were different. This difference in the plating efficiencies for the different deposited materials was attributed to the simultaneous occurrence of an unintended reduction reaction on the cathode (AgNW) surface: a hydrogen gas generation reaction [51–53]. This side reaction was accompanied by a reduction reaction of the solvated metal ions, which competitively suppressed the deposition of the target material completely on the cathode (AgNW) surface. The overpotential of the hydrogen evolution reaction is different for different materials. The overpotential of hydrogen evolution for Ni is largest among those (Ni: 0.70 V, Cu: 0.45 V, Pd: 0.24 V) for used electroplating materials; [54–56] as a result, highest amount of hydrogen gas is generated, thus the electroplating rate can be greatly reduced without vigorous reaction. This is in good agreement with the result from previous studies that the plating efficiency of PdCl-based Pd electroplating was reported to be higher than 97%, and the plating efficiencies in the case of using Cu- and Ni-based electroplating solutions were reported to be much lower values of 95% and 90%, respectively [57–59]. Therefore, Ni-Ag NWs could be the best electroplating system having a smooth surface by preventing over-growth of nickel through the competitive reaction and thereby suppressing the uneven surface of the nickel layer [39]. In addition, the cross-sectional high-resolution transmission electron microscopy (HR-TEM) image and energy dispersive X-ray spectrometry (EDS) line analysis of Ni-AgNW in Additional file 1: Fig. S2 demonstrated the successful electroplating of Ni in the shell area.

Sheet resistance of the M-AgNWs was monitored as a function of electroplating time by the four-point probe technique, as shown in Fig. 3b. The pristine AgNW electrode showed a sheet resistance of 19 Ω/sq. As the electroplating time increased, sheet resistances of both the Ag-AgNWs and Ni-AgNWs gradually reduced from 19 Ω/sq to 3 Ω/sq and to 9 Ω/sq, respectively. When the nanowires were deposited on the substrate, high electrical resistance was generated at the nanowire junctions. The electroplated metal filled the nano-gap at the nanowire junctions, which reduced the high electrical resistance. However, there was a difference in the trend of change in sheet resistances of the Cu-AgNWs and Pd-AgNWs. The sheet resistance of the Cu-AgNWs increased considerably up to 33 Ω/sq in the early stage of the electroplating process (less than 2 s of electroplating time) and then gradually decreased to 8 Ω/sq with a further increase in the electroplating time. The sheet resistance of the Pd-AgNWs fluctuated during the electroplating process; it increased up to an electroplating time of 6 s and then decreased with a further increase in the electroplating time to 8 s, and it subsequently increased to 37 Ω/sq at the end of the electroplating process (i.e., at 20 s). In general, during the deposition of a given metal on AgNWs, a contact resistance is generated between the two metals (i.e., Ag and the deposited metal), which increases the sheet resistance of the resultant electrode [38, 60]. Both Cu-AgNWs and Pd-AgNWs (green and blue arrows, respectively, in Fig. 3b) showed an increase in sheet resistance in the early stage of electroplating. When Pd was plated for 10 s or longer, the sheet resistance increased again because of non-uniform metal growth [61, 62]; therefore, the NW surface became rougher with an increase in the electroplating time from 8 to 20 s (see Fig. 2d).

The optical transmittance was also monitored throughout the electroplating process (Fig. 3c). The transmittance of the pristine AgNW electrode was 92% at 550 nm (Additional file 1: Fig. S3). For all the electroplated NW network electrodes, the optical transmittance decreased with an increase in electroplating time; the optical transmittances at 20 s (i.e., the end of electroplating) were 37%, 59%, 49%, and 30%, respectively, for the Ag-AgNW, Ni-AgNW, Cu-AgNW, and Pd-AgNW electrodes. As the thickness of the core–shell nanowires increased, the distance between the nanowires decreased which reduced the transmission of visible light through core–shell nanowires electrode. The Pd-AgNW electrode showed the lowest optical transmittance despite having high sheet resistance although the low transmittance TCE typically showed low sheet resistance. The Ni-AgNW electrode showed the highest transmittance at an electroplating time of 20 s. Figure 3d shows the optical transmittance of the M-AgNW films as a function of sheet resistance. For a metal-electroplated NW electrode to be applicable as an anode of OLEDs, the electrode should have a high transparency exceeding 80% even when its resistance is higher than expected. For comparison purposes, the following four M-AgNW network electrodes with optical transmittances of ~ 80% were selected for OLED fabrication: Ag-AgNW electrode (9 Ω/sq), Ni-AgNW electrode (15 Ω/sq), Cu-AgNW electrode (27 Ω/sq), and Pd-AgNW electrode (43 Ω/sq).

Metal electroplating increased the WF of the AgNW electrode in addition to reducing its sheet resistance. Hole injection from the anode to the overlying organic layers should be improved for achieving higher current efficiency of OLEDs. In this study, hole injection from the pristine AgNW anode to the organic layer was insufficient because of the high hole-injection energy barrier (~ 0.8 eV) at their junction (Additional file 1: Fig. S4). UV photoelectron spectroscopy (UPS) measurements were performed to estimate the WFs of the metal-electroplated NWs, as shown in Figure S5. The WFs of the Ag-AgNWs, Ni-AgNWs, Cu-AgNWs, and Pd-AgNWs were measured to be 4.2, 5.0, 4.6, and 4.8 eV, respectively. These values accurately corresponded to the literature-reported values for these metals (4.3, 5.0, 4.7, and 4.8 eV for Ag, Ni, Cu, and Pd, respectively). Precise control of the WF through metal electroplating of the AgNWs effectively lowered the hole-injection barrier at the junction between the M-AgNW electrode and the overlying organic layer. Furthermore, the junction welding caused by electroplating dramatically improved the mechanical stability of the electrode as shown in Additional file 1: Fig. S6.

**Fig. 4:**
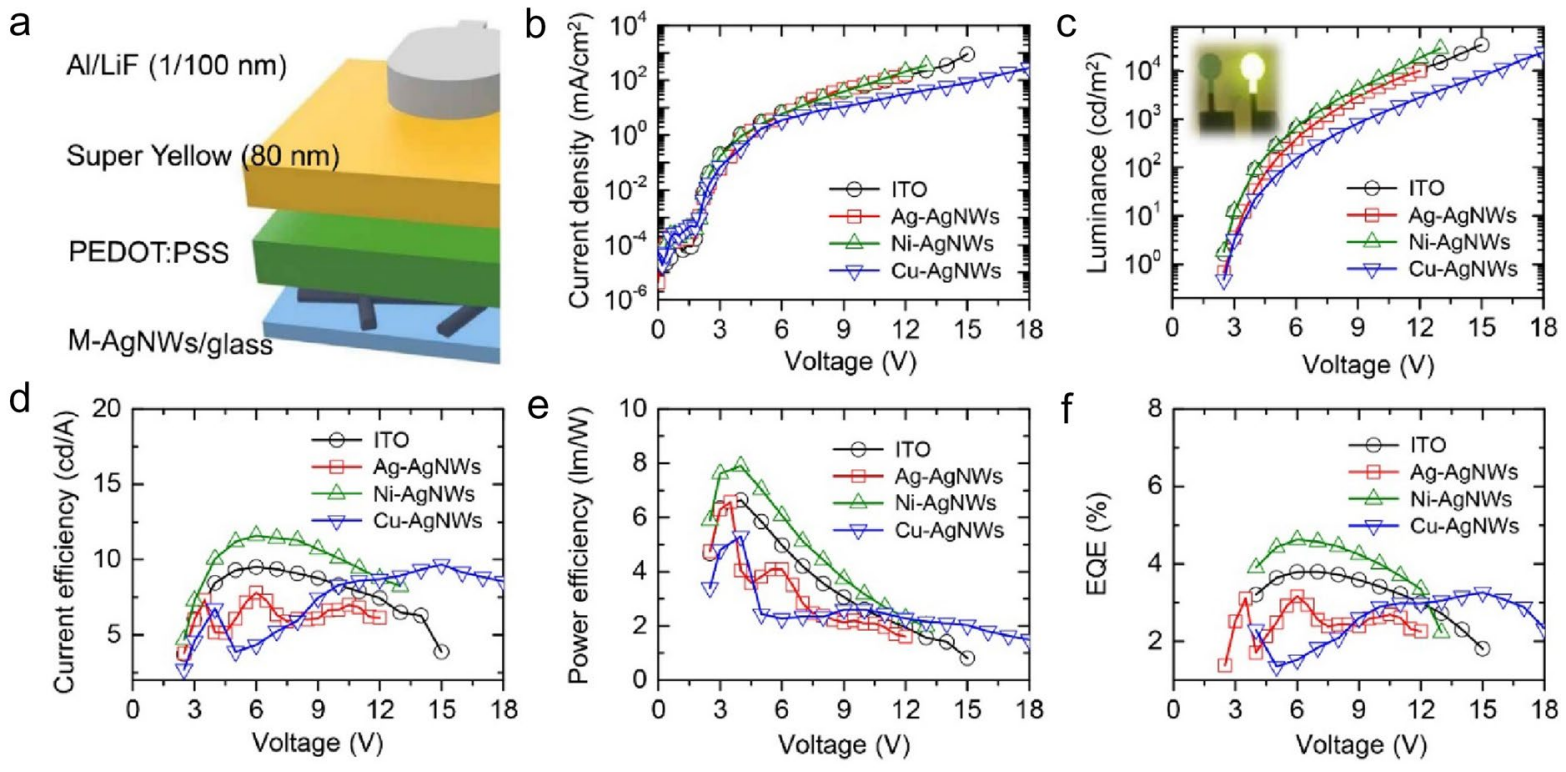
**a** Schematic structure of OLED with M-AgNW anode. **b** Current density and **c** luminance characteristics of OLEDs with ITO and M-AgNW anodes as functions of applied voltage. The inset shows a photographic image of a light-emitting pixel of an OLED. **d** Current efficiency, **e** power efficiency, and **f** external quantum efficiency (EQE) of OLEDs

Finally, OLEDs with the prepared M-AgNWs as their anodes were fabricated in a glass/M-AgNW/PEDOT:PSS (50 nm)/Super Yellow (80 nm)/LiF (1 nm)/Al (100 nm) structure, as shown in Fig. 4a. Only three types of metal electroplated nanowires such as Ag-AgNWs, Ni-AgNWs, and Cu-AgNWs based OLEDs were fabricated because high sheet resistance of Pd-AgNWs is not suitable for OLED fabrication. From the current density–voltage–luminance characteristics (Fig. 4b and c), it was found that the Ni-AgNW OLED had a higher current density and lower turn-on voltage (*V*_ON_, 2.45 V) than that of the Ag-AgNW OLED (2.62 V) and Cu-AgNW OLED (2.70 V) (Additional file 1: Table S1). This result indicated that an increased WF of the Ni-AgNW electrode corresponded to a lower hole-injection barrier at the anode/PEDOT:PSS interface, which facilitated hole injection into the emitting layer. Furthermore, the Ni-AgNW OLED (11.60 cd/A; 7.90 lm/W; 4.63%; 28,564 cd/m^2^) had considerably better efficiencies and luminance than the Cu-AgNW OLED (9.68 cd/A; 5.30 lm/W; 3.27%; 23,813 cd/m^2^) and Ag-AgNW OLED (7.80 cd/A; 6.56 lm/W; 3.17%; 9,908 cd/m^2^) because of the low hole-injection barrier (Fig. 4d–f). Specifically, the efficiency of the Ni-AgNW OLED was higher than even that of an ITO-based OLED (9.51 cd/A; 6.64 lm/W; 3.80%; 34,218 cd/m^2^) because of the optimized and hence lowest hole-injection barrier and relatively lower sheet resistance of the Ni-AgNW electrode. In addition, compared to ITO-based PLEDs in which about 20% of light is typically trapped by the waveguide mode, AgNWs-based PLEDs can have improved outcoupling efficiency from the scattering effect and plasmonic effect on the nanowire surface with much higher theoretical maximum efficiency.

## Conclusion

To summarize, we demonstrated metal (Ag, Ni, Cu, or Pd)-electroplated core–shell AgNWs and their application as the anode electrode of OLEDs. The solvated metal ions (Ag^+^, Ni^2+^, Cu^2+^, and Pd^2+^) in the respective electroplating baths were transformed into the corresponding metals on the AgNW surface by electrochemical reduction. The amount of metal (shell) electroplated on the AgNWs (core) was deterministically controlled by varying the electroplating conditions. Ni electroplating increased the WFs of the AgNWs, which, in turn, facilitated hole injection at the AgNW–PEDOT:PSS junction and decreased the sheet resistance of the AgNWs; as a result, OLEDs having these metal-electroplated AgNWs as their anode electrodes showed enhanced overall luminance characteristics. This simple and controllable metal electroplating method for increasing the WFs and electrical conductivities of AgNW electrodes provides significant scope for innovation in the development of TCEs for next-generation optoelectronic devices.

## Supplementary Information

**Additional file 1:** **Figure S1.** (a) SEM and (b) AFM images of as-coated (pristine) AgNW film. **Figure S2.** Cross-sectional HR-TEM image and (b) EDS line analysis of Ni-AgNW. **Figure S3.** Optical transmittance of as-coated AgNW film as a function of wavelength. **Figure S4.** Energy band structure of OLED with M-AgNW anode. **Figure S5.** UPS spectra of M-AgNW films. **Figure S6.** *R*/*R*_0_ of the Ni-AgNWs as a function of the fatigue cycle (tensile strain = 2.0 %). *R* is the sheet resistance and *R*_0_ is the initial sheet resistance. **Table S1.** Turn-on voltage and each operating voltage at 10nit, 100nit, 1000nit 10,000nit of PLEDs with different anodes.

## Data Availability

Not applicable.
