# Supplementary material for: Electroplated core–shell nanowire network electrodes for highly efficient organic light-emitting diodes
Source: Nano Converg. 2022 Jan 5;9:1. doi: 10.1186/s40580-021-00295-2 (PMC8733141; doi:10.1186/s40580-021-00295-2)
Supplement: Supplementary file 1 — Additional file 1: Figure S1. (a) SEM and (b) AFM images of as-coated (pristine) AgNW film. Figure S2. Cross-sectional HR-TEM image and (b) EDS line analysis of Ni-AgNW. Figure S3. Optical transmittance of as-coated AgNW film as a function of wavelength. Figure S4. Energy band structure of OLED with M-AgNW anode. Figure S5. UPS spectra of M-AgNW films. Figure S6. R/R0 of the Ni-AgNWs as a function of the fatigue cycle (tensile strain = 2.0 %). R is the sheet resistance and R0 is the initial sheet resistance. Table S1. Turn-on voltage and each operating voltage at 10nit, 100nit, 1000nit 10,000nit of PLEDs with different anodes. [file 40580_2021_295_MOESM1_ESM.docx]

Supporting information for

**Electroplated Core–Shell Nanowire Network Electrodes for Highly Efficient Organic Light-Emitting Diodes**

Hyungseok Kang,^1,†^ Joo Sung Kim,^3,†^ Seok-Ryul Choi,^2,†^ Young-Hoon Kim,^3^ Do Hwan Kim,^4^ Jung-Gu Kim,^2^ Tae-Woo Lee,^3,5^* Jeong Ho Cho^6,^*

^1^SKKU Advanced Institute of Nanotechnology (SAINT), ^2^School of Advanced Materials Science and Engineering, Sungkyunkwan University, Suwon 440-746, Republic of Korea.

^3^Department of Materials Science and Engineering, Seoul National University, Seoul 08826, Republic of Korea

^4^Department of Chemical Engineering, Hanyang University, Seoul 04763, Republic of Korea

^5^School of Chemical and Biological Engineering, Research Institute of Advanced Materials, Institute of Engineering Research, Nano Systems Institute (NSI), BK21 PLUS SNU Materials Division for Educating Creative Global Leaders, Seoul National University, Seoul 08826, Republic of Korea

^6^Department of Chemical and Biomolecular Engineering, Yonsei University, Seoul 03722, Republic of Korea.

^†^These authors contributed equally to this work.

*Corresponding authors: [jhcho94@yonsei.ac.kr](mailto:jhcho94@yonsei.ac.kr), [twlees@snu.ac.kr](mailto:twlees@snu.ac.kr)


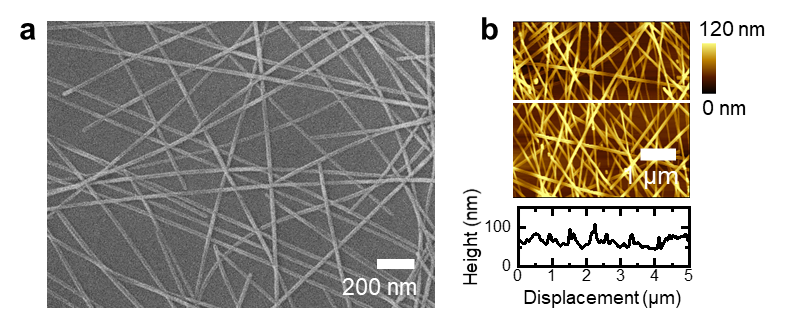


**Figure S1.** (a) SEM and (b) AFM images of as-coated (pristine) AgNW film.


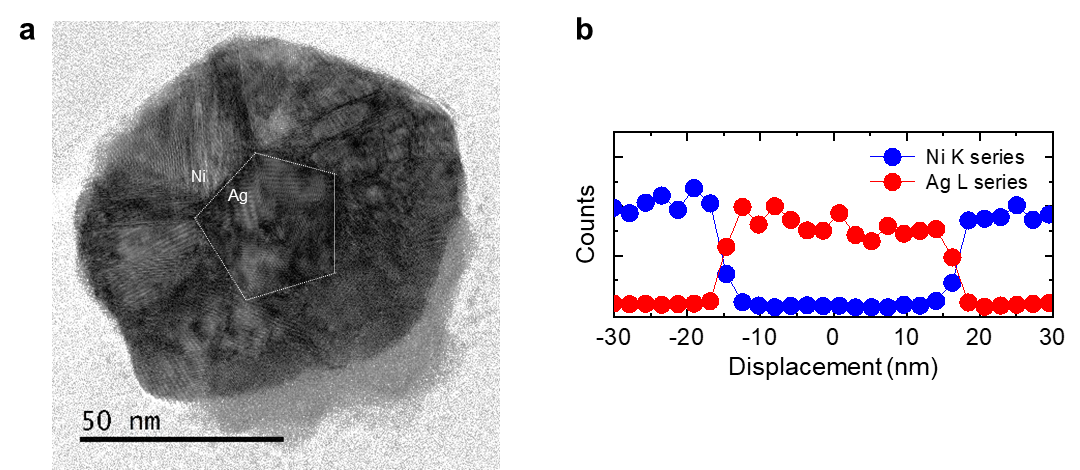


**Figure S2.** Cross-sectional HR-TEM image and (b) EDS line analysis of Ni-AgNW.


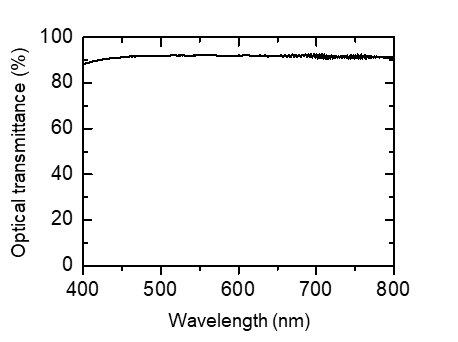


**Figure S3.** Optical transmittance of as-coated AgNW film as a function of wavelength.


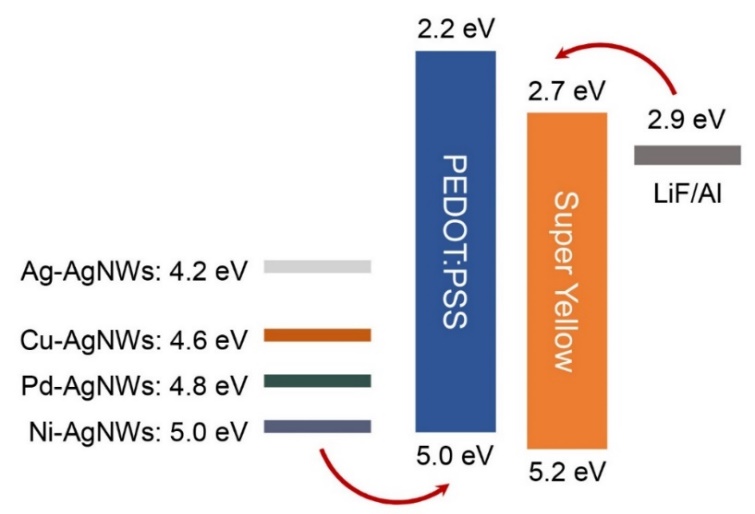


**Figure S4.** Energy band structure of OLED with M-AgNW anode.


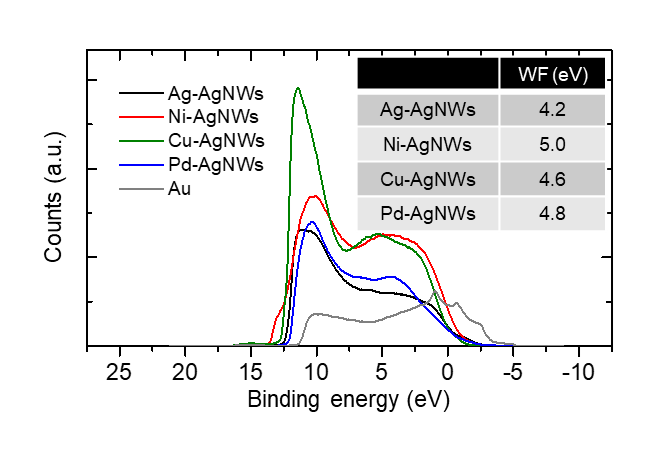


**Figure S5.** UPS spectra of M-AgNW films.


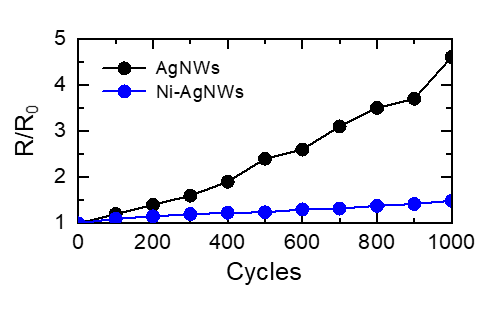


**Figure S6.** *R*/*R*_0_ of the Ni-AgNWs as a function of the fatigue cycle (tensile strain = 2.0 %). *R* is the sheet resistance and *R*_0_ is the initial sheet resistance.

**Table S1.** Turn-on voltage and each operating voltage at 10nit, 100nit, 1,000nit 10,000nit of PLEDs with different anodes.

|  | Turn-on voltage  (@ 1 nit) | Voltage  @10 nit | Voltage  @ 100 nit | Voltage  @ 1,000 nit | Voltage  @ 10,000 nit |
| --- | --- | --- | --- | --- | --- |
| Ni-AgNW | ~2.4V | ~2.94 V | ~4.082 V | ~6.547 V | ~10.8 V |
| ITO | ~2.4V | ~2.96 V | ~4.082 V | ~6.7 V | ~11.77 V |
| AgNW | ~2.62 V | ~3.41 V | ~4.728 V | ~7.27 V | ~12.05 V |
| Cu-AgNW | ~2.7 V | ~3.59 V | ~5.5 V | ~9.51 V | ~15.7 V |
